# Intelligent tuning method for service scheduling in electric power communication networks based on operational risk and QoS guarantee

**DOI:** 10.1371/journal.pone.0317564

**Published:** 2025-02-24

**Authors:** Yang Yu, YueLin Jiang, Zeng Dou, Li Cong, Wei Huang, Qiang Zhang, Yang Hu, YanJun Bi

**Affiliations:** 1 The State Grid Jilin Province Electric Power Company Limited Information Communication Company, Jilin, China; 2 Changchun University of Science and Technology High-tech Industry Company Limited, Jilin, China; 3 State Grid Changchun Electric Power Supply Company, Jilin, China; Northwestern Polytechnical University, CHINA

## Abstract

In the operational planning of electric power communication networks, a well-structured service scheduling scheme based on the established network topology can significantly enhance the risk prevention capabilities of these networks. Since routing policies directly influence data transmission paths, routing optimization serves as an effective strategy for improving network performance by mitigating transmission risks and threats. This paper introduces an Intelligent Tuning Method for Service Scheduling in Electric Power Communication Networks Based on Operational Risk and Quality of Service (QoS) Guarantee. Based on a comprehensive assessment of service transmission reliability and time costs, a route satisfaction evaluation function model has been developed. Utilizing this model, an enhanced Risk-Time Ant Colony Optimization (RT-ACO) routing algorithm is proposed, which builds upon the traditional ant colony algorithm. The improvements to the ant colony algorithm are made in four key areas: the definition of heuristic information, the weighting of parameters, the state selection strategy, and the pheromone update strategy. These enhancements aim to achieve optimal routing scheduling based on risk information. At the same time, a reconfiguration algorithm for power optical communication networks, based on service priority, is proposed for specific service requests. This algorithm provides both a primary routing path and an alternate routing path for service transmission, ensuring the delivery of high-priority services even when both the primary and standby paths are unavailable. Simulation results from an actual power business communication network demonstrate that the algorithm outputs the main and alternate paths with the lowest risk costs. Additionally, the path satisfaction of the proposed algorithm is improved by 7.4% compared to the traditional ant colony algorithm. This improvement validates the accuracy and superiority of the proposed algorithm and offers a valuable reference for ensuring the reliable operation of power optical fiber communication network systems.

## 1. Introduction

Risk optimization is the cornerstone of a network risk management system, encompassing the identification and assessment of potential risks, as well as the formulation of appropriate protective measures. By employing intelligent analysis of potential risks within the communication network, early warning systems and emergency management strategies can be implemented to ensure the safe operation of the network. Among the various risk mitigation methods, route optimization can be executed without altering the physical network topology, thereby enhancing the overall performance, stability, and security of the network [1,2].

Currently, there are several methods to optimize risk, including route-based optimization [3–10], topology-based optimization [11,12], load balancing optimization based on demand response services [13], and optimization focused on network service quality and trustworthiness [14]. In [14], a heuristic function was developed based on bandwidth and trust value, taking into account network service quality and trustworthiness, with the goal of enhancing the success rate of re-routing during network failures. However, this approach did not differentiate between services. In [6], optimization was approached from the perspective of service performance by introducing the concept of a virtual queue, which aimed to optimize delay and packet loss rates. Nevertheless, the evaluation metric was limited to service utility. In [15], a path optimization mechanism based on the traditional ant colony algorithm was proposed for optical networks, but it did not optimize the key parameters of the algorithm or clarify the heuristic information used. In [7], the objective function of the routing optimization strategy was enhanced by incorporating bandwidth utilization rate, bandwidth utilization rate grade, and transmission delay, based on predictions of link traffic changes. This enriched the optimization evaluation metrics. In [16], a fault recovery method was proposed for power communication networks, focusing on the identification of critical information nodes. In [8], routing allocation was based on service grade, and a link reliability function model was established from the perspective of service reliability. The importance of service and link parameters was calculated using the analytic hierarchy process. In [9], the routing path selection criteria considered both delay and node importance; however, node importance was calculated based on network topology, and the factors considered lacked comprehensiveness. In [17], a task scheduling algorithm based on multi-QoS constraint optimization was implemented, with the optimization goal defined by the combined influence of time, reliability, and security factors. In [18], the optimization objective was to identify the path with the minimum energy consumption, which differed from previous approaches and utilized a combination of immune clonal and frog-hopping algorithms.

According to the preceding discussion, most existing studies have overlooked the risk modeling aspects of business reliability and network topology reliability. Additionally, they have failed to consider the delays incurred when business packets must wait in line after arriving at the same routing node. In path planning algorithms, the majority employ intelligent biomimetic algorithms, with improvements typically achieved by combining two such algorithms. Consequently, the risk optimization method proposed in this paper primarily concentrates on modeling the optimal objective function and designing routing optimization algorithms.

## 2. Routing optimization strategy and mathematical model

This chapter primarily introduces the optimization model for routing in power optical communication networks. It also discusses the reliability model of power optical communication networks, the power service forwarding delay model, and the routing optimization model. The chapter focuses on the routing optimization problem within power communication networks, with the communication mode of the nodes configured to utilize optical fiber technology.

### 2.1. Network model of power optical communication network

**Fig 1:**
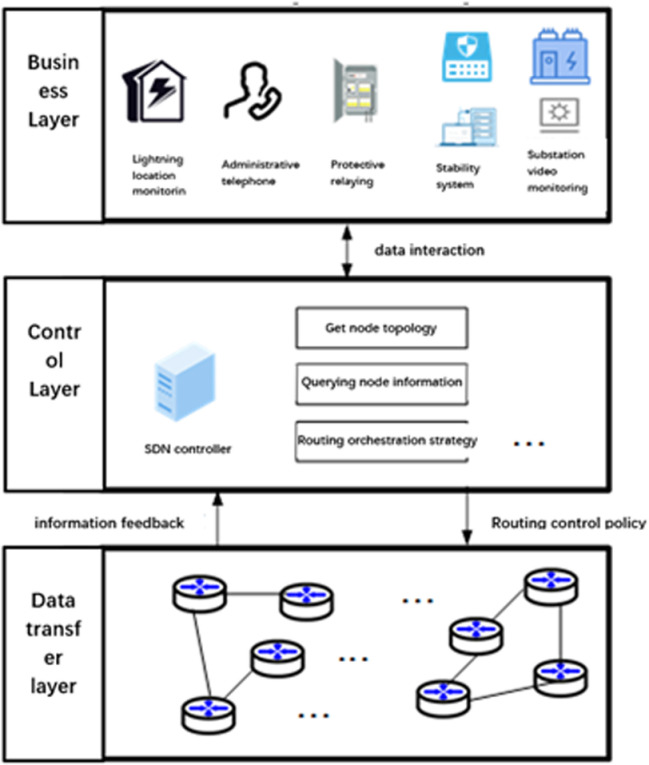
Network architecture of electric power communication network.

The network architecture of a power communication network based on Software-Defined Networking (SDN) technology is illustrated in Fig 1. This architecture comprises three layers: the business layer, the control layer, and the data transmission layer, arranged from top to bottom. Data interaction occurs between the service layer and the SDN control layer. The service layer encompasses various types of services, each with different priorities. The control layer houses the SDN controller. A defining characteristic of SDN is the separation of data and control, which enables the system to manage information from diverse service types while also acquiring topology information for the entire network within the data transmission layer. The data transmission layer provides routing topology information to the control layer and allows for direct control functions by the control layer.

Graph *G* is used to represent the network model of power optical communication network, G=(V,E), where *V* is the node set and *E* is the edge set. At the same time, if the number of nodes in the graph *G* is *N* and the number of edges is *L*, then the node set is represented by: $V=\left\{v_{1},v_{2},\cdots ,v_{i},\cdots ,v_{N}\right\}$, and the edge set is represented by: $E=\left\{e_{1},e_{2},\cdots ,e_{g},\cdots ,e_{L}\right\}$. Among them, i=1,2,⋯,N, g=1,2,⋯,L. Defines $S=\left\{s_{1},\cdots ,s_{r},\cdots ,s_{R}\right\}$ representing the business data source node, and S⊂V. In addition, in the time modeling and calculation, this paper adopts *T* discrete time model that subdivides the overall optimization cycle into equal-length time slots, expressed as $T=\left\{1,\cdots ,t,\cdots T\right\}$, the length of each time slot is $\tau_{0}$. The source node generates power service packets at the beginning of each time slot according to the specific service requirements and time scheduling. It is forwarded to the destination node of the power service through multiple intermediate nodes with multiple hops. It is defined R(s,f) as a routing request from the source node *s* to the destination node *f*. Any path in Graph *G* is a set of X={s→...i→f}, x∈X indicates a certain communication link.

### 2.2. Power service forwarding delay model

Since the propagation speed of the digital signal in the optical fiber is 2∗10^8 m/s, the propagation delay on the communication link is relatively small and can be ignored. The performance of hardware devices such as hosts or routers is assumed to be consistent, so the processing delay caused by processing data is not considered. The emphasis is on sending delay and queuing delay. According to the queuing theory, at the *t* time slot, the power service packets from different source nodes are defined as $P^{t}=\left\{P_{1}^{t},\cdots ,P_{s}^{t},\cdots P_{S}^{t}\right\}$, the packet $P_{s}^{t}$ size is $B_{s}^{t}$. The nodes that pass through from the source node are $z_{s}^{t}$, and are formed $Z_{s}^{t}$. When the packet $P_{s}^{t}$ is located on the routing node $z_{s}^{t}$ and the next hop routing node selected is $v_{i}$ ($v_{i}\neq z_{s}^{t}$), Let $Z_{s}^{t}=Z_{s}^{t}\cup (z_{s}^{t}+1=v_{i})$, then the element in $Z_{s}^{t}$ satisfy. $z_{s}^{t}\in V$, $1_{s}^{t}=s_{r}$.

The data transmission rate supported by the power communication network is defined as *R*, and the transmission (sending) delay $T_{r}(z_{s}^{t})$ of data packets $P_{s}^{t}$ at any routing node is defined as:


$$T_{r}(z_{s}^{t})=\frac{B_{s}^{t}}{R}$$ (1)

Due to the limited data processing capacity of the routing node, based on the principle of first come first service, when the current packet has not finished forwarding on the node, the subsequent packets will be placed in the buffer to wait. Subsequent packets are not transmitted until the previous packets have been forwarded. Sets the indicator variable $\gamma (c_{i}^{t},z_{s}^{t})\in \left\{0,1\right\}$, which $\gamma (c_{i}^{t},z_{s}^{t})=1$ indicates that at the *t* time slot, the node $v_{i}$ forwards the $c_{i}^{t}$ packet that is the same as the packet $P_{s}^{t}$ transmitted to the routing node $z_{s}^{t}$, otherwise $\gamma (c_{i}^{t},z_{s}^{t})=0$.

This paper stipulates that each routing node can only forward one packet at a time, and a single packet can only be forwarded on one routing node at the same time, Therefore, the queuing delay $T_{Q}(z_{s}^{t})$ of power service packets $c_{i}^{t}$ at the routing node $z_{s}^{t}$ is:


$$T_{Q}(z_{s}^{t})=\sum_{v_{i}\in V}\sum_{c_{i}^{t}\in N^{+}}\gamma (c_{i}^{t},z_{s}^{t})T_{Q}(c_{i}^{t})$$ (2)

In summary, the time *T* taken by the next hop routing node of the power service packet $P_{s}^{t}$ is:


$$T=T_{r}(z_{s}^{t})+T_{Q}(z_{s}^{t})$$ (3)

### 2.3. Reliability model of power optical communication network

The status of communication services can be categorized into four distinct situations:

(1) Normal: The active and standby routes for services remain unaffected, and communication requirements can be fulfilled.
(2) The active route is impacted: The active service route is interrupted and switched to the standby route.
(3) The standby route is affected: The standby route for services has been interrupted.
(4) Interrupt state: When both the primary and alternate routing paths for service transmission are unavailable, the service transmission is interrupted, resulting in a loss of communication capability.

Based on the content of the first two chapters, a risk model is established that takes into account the characteristics of nodes within the network's physical topology, service capacity, and resource utilization.

*Q* is set as the quantitative value of the severity of risk consequences. According to the indicator system of “static structure-dynamic operation”, $q_{1}$ is the influence value of the static structure of the communication network, and $q_{2}$ is the influence value of the dynamic operation status.


$$\begin{array}{l} Q=q_{1}+q_{2}\\ \;=\sum_{j=1}^{m_{1}}w_{j}F\left(U_{j}\right)+\sum_{j=m_{1}}^{m}w_{j}F\left(U_{j}\right) \end{array}$$ (4)

Where, $F\left(U_{j}\right)$ represents the value of each evaluation index and $w_{j}$ is the weight of the index *j*. For cost-based indicators, $F\left(U_{j}\right)$ is the sum of the risk values of each indicator. For the benefit indicator, the deviation of the indicator is used to measure the index risk value, and the calculation function of $F\left(U_{j}\right)$ is as follows:


$$F\left(U_{j}\right)=\sum_{z=1}^{N_{n}}\frac{e^{\max\left(\left|\frac{V_{j}-{\bar{V}}_{j}}{{\bar{V}}_{j}}\right|,\left|\frac{{\underline{V}}_{j}-V_{j}}{{\underline{V}}_{j}}\right|,0\right)}-1}{e-1}$$ (5)

Where, $N_{n}$ is the total number of unit nodes in the network, $V_{j}$ is the value of nodes *Z* under the indicator *j*, ${\bar{V}}_{j}$ and ${\underline{V}}_{j}$, respectively, are the upper and lower limits of nodes *Z* under the indicator *j* measurement.

Set Risk as the sum of the risk values of each node unit. According to formula (4) and (5), the reliability model of the power optical communication network is established as formula (6):


$$Risk=\sum_{j=1}^{m_{1}}w_{j}F\left(U_{j}\right)+\sum_{j=m_{1}}^{m}w_{j}F\left(U_{j}\right)$$ (6)

### 2.4. Power optical communication network routing optimization problem model

In this paper, we primarily consider two objectives in the formulation of a routing optimization strategy: Quality of Service (QoS) and the operational risk value of power optical communication networks. This strategy can be divided into two scenarios. First, the transmission path for power services is optimized under normal conditions, providing both a primary routing path and an alternate routing path for service transmission to mitigate risks. Second, when the active and standby communication routing links fail, services are restored based on priority to ensure the timely transmission of critical scheduling information. This approach involves implementing effective service scheduling and resource allocation to minimize information delays and transmission risks.

To measure the influence of each risk factor, this paper defines the path satisfaction index. The objective function for the path *x* is defined as follows:


$$\begin{array}{l} \max F(x)=\alpha_{1}\frac{Risk_{\max}-Risk(x)}{Risk_{\max}}+\alpha_{2}\frac{T_{\max}-T(x)}{T_{\max}}\\ \text{ s}.\text{t}. \{\begin{array}{c} \alpha_{1}+\alpha_{2}=1\\ \alpha_{1},\alpha_{2}>0\\ T\leq T_{\max}\\ Risk\leq Risk_{\max}\\ B\geq B_{\min} \end{array} \end{array}$$ (7)

When the system receives a power communication service request R(s,f), it can find the optimal solution that maximizes the value of the objective function F(x) by applying the appropriate optimization algorithm to solve the objective function of each path and ensure that the constraint conditions are met.

## 3. Route optimization algorithm design

This chapter primarily presents the design of a routing optimization algorithm for power optical communication networks. It discusses the traditional ant colony algorithm, the routing optimization algorithm utilized in this study, and its enhancements. Furthermore, it introduces a routing reconstruction algorithm for power optical communication networks that is based on service priority.

### 3.1. Traditional ant colony algorithm

In order to facilitate understanding of the algorithm, this section introduces the fundamental processes and principles of Ant Colony Optimization. It also describes the functions involved in this optimization technique and their corresponding parameters, as illustrated in Table 1.

**Table 1:** Ant colony algorithm function and parameter explanation.

Symbol	Definition
*m*	The number of ants in a colony
$b_{i}(t)$	The number of ants at node i at time t
$d_{ij}$	The distance between two nodes i and j
$\eta_{ij}$	Heuristic function
$\tau_{ij}$	Pheromone trace intensity on the edge (i,j)
$\Delta \tau_{ij}$	The amount of trace pheromone per unit length left by ant k on an edge (i,j)
$P_{ij}^{k}$	The transfer probability of ant k
*ρ*	The persistence of pheromone tracks
1−ρ	Attenuation coefficient of pheromone locus (Volatilization coefficient)
*α*	The relative importance of pheromone trajectories
*β*	The relative importance of the degree of inspiration
*Q*	Pheromone concentration

Where, $m=\sum_{i=1}^{n}b_{i}(t)$.

#### 1. The ant colony algorithm primarily consists of the following stages.

**Fig 2:**
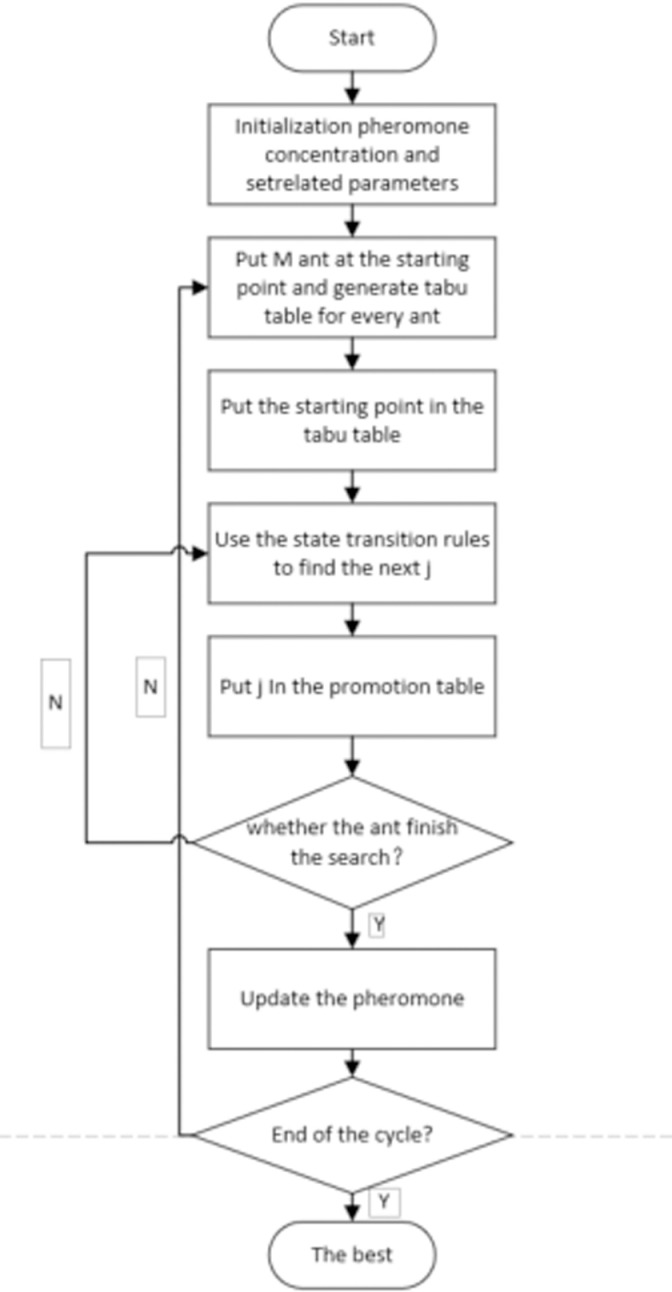
Flowchart of simple ant colony algorithm.

The flow of the simple ant colony algorithm is illustrated in Fig 2. First, the parameters are initialized, and the fitness of each ant is evaluated based on the objective function. Subsequently, the pheromone levels on the paths are updated according to the fitness values of the ants. Ants with higher fitness typically deposit more pheromones on their paths, thereby attracting other ants to select those routes.

#### 2. Algorithm description.

Set $\tau_{ij}(0)=C$ (*C* is constant), that is, the pheromone concentrations on all paths are set to equal initial values. Ant *k* (k=1,2,⋯,m) determines the direction of diversion based on the amount of pheromone on each path. The ant system uses random proportional rules to determine the probability that ants move between nodes. Assuming that the ant *k* is located at the node *i* at time *t*, the transfer probability $P_{ij}^{k}$ of it choosing to move to the node *j* is expressed as follows:


$$P_{ij}^{k}= \{\begin{array}{c} \frac{\tau_{ij}^{a}(t)\eta_{ij}^{\beta }(t)}{\sum_{s\in allowed_{k}}\tau_{ij}^{a}(t)\eta_{ij}^{\beta }(t)},j\in allowed_{k}\\ 0,\;,otherwise \end{array}$$ (8)

Where, $allowed_{k}=\left\{0,1,\cdots ,n-1\right\}$ indicates the node that can be selected by the ant *k* in the next step. According to the above formula, the transition probability $P_{ij}^{k}$ is proportional to $\tau_{ij}^{a}(t)\cdot \eta_{ij}^{\beta }(t)$. The pheromone concentration on each path was adjusted according to formulas (9) and (10).


$$\tau_{ij}(t+1)=\rho \cdot \tau_{ij}(t)+\text{Δ}\tau_{ij}(t,t+1)$$ (9)

$$\text{Δ}\tau_{ij}(t,t+1)=\sum_{k=1}^{m}\text{Δ}\tau_{ij}^{k}(t,t+1)$$ (10)

Where, $\text{Δ}\tau_{ij}^{k}(t,t+1)$ denotes the amount of pheromone released by the *k* ant along the path (i,j). If ants excel on a certain path, they usually release more pheromones. $\text{Δ}\tau_{ij}(t,t+1)$ indicates the pheromone increment of the cycle path (i,j).

M. Dorigo once proposed three different pheromone renewal models: ant cycle system, ant quantity system and ant density system [19].

In the ant cycle system model


$$\text{Δ}\tau_{ij}^{k}(t,t+1)= \{\begin{array}{cc} \frac{Q}{L_{k}} & \text{If the k ant passes through path }(i,j)\text{ in this cycle}\\ 0 & otherwise \end{array}$$ (11)

In the other two models, the difference from the above system model is only the difference in the expression of $\text{Δ}\tau_{ij}^{k}(t,t+1)$ under the passing path (i,j) condition. This paper selects the ant cycle model which can update the whole information. Where, $L_{k}$ is the length of the path passed by the *k* ant. According to the specific research background and problems, the expression form of, $\text{Δ}\tau_{ij}$、 $\text{Δ}\tau_{ij}^{k}$ and $P_{ij}^{k}$ can be redefined and adjusted.

### 3.2. Power optical communication network routing optimization algorithm based on path satisfaction

Route optimization is an effective method that maintains the original network topology. Building on the risk assessment results presented in the previous chapter, this chapter develops a route selection model based on path satisfaction to ensure the normal operation of the power optical communication network. Additionally, it introduces the RT-ACO (Risk-Time Ant Colony Optimization) route optimization algorithm. This algorithm enhances the heuristic function of the ant colony model by incorporating both risk and delay costs. Furthermore, the algorithm's parameters are optimized, and the selection strategy is refined.

#### 1. RT-ACO (Risk-Time-ACO) algorithm description.

In this paper, the packet is analogous to an ant, which is dispatched from a network node by applying a probabilistic transfer rule. It is then forwarded by intermediate nodes until it reaches the destination node, thereby establishing a path from the source node to the final node. Furthermore, when employing the traditional ant colony algorithm to address the routing optimization problem, four modules of the ant colony algorithm have been enhanced.

(1) Improvement of heuristic function of ant colony model, which has a new definition, which no longer refers to the crawling length (path length) of “ant” alone, but includes the delay and risk cost of “ant” in the process from node *i* to node *j*, including the comprehensive heuristic function of multiple factors.


$$\eta_{ij}=Risk_{one}+T_{one}$$ (12)

Since the risk and delay indexes have different dimensions and their values are quite different, the two indexes are normalized respectively. $Risk_{one}$ is the normalized value of path risk cost; $T_{one}$ normalized value for link cost time.


$$\bar{Risk}=\frac{\sum_{x\in R(s,f)}Risk}{s\to f\text{Number of links}}$$ (13)

$$\bar{T}=\frac{\sum \left(T(s)+\cdots +T(f)\right)}{s\to f\text{Number of nodes}}$$ (14)

$\bar{Risk}$ is the average risk cost of the path; $\bar{T}$ is the average delay cost of the path. $Risk_{\max}^{s\to f}$, $Risk_{\min}^{s\to f}$ respectively represent the maximum and minimum value of the path risk cost; $T_{\max}$, $T_{\min}$ respectively are the maximum and minimum values of the delay cost in the link, and then their normalized values are calculated.


$$Risk_{one}=\frac{\bar{Risk}-Risk_{\min}^{s\to f}}{Risk_{\max}^{s\to f}-Risk_{\min}^{s\to f}}$$ (15)

$$T_{one}=\frac{\bar{T}-T_{\min}}{T_{\max}-T_{\min}}$$ (16)

(2) Genetic algorithm is used to optimize the parameter *α* and *β* in equation (6). In the process of path selection, ants will be guided according to the intensity of information left on the path and the link cost between nodes, and the parameter *α* and *β* determines the relative weight of pheromone and path cost. However, no universal mathematical analysis method has been identified that guarantees the generation of optimal parameter settings in every scenario. Historically, statistical data for parameters were obtained through manual selection based on experience or through step-by-step simulations using cyclic combination enumeration. However, this approach inevitably leads to some deviation in the empirical values for different problems. In such cases, it is more effective to seek the optimal solution for the hyper parameters in proximity to the empirical values. Therefore, this paper employs a genetic algorithm to optimize the key parameters of the ant colony algorithm, specifically targeting the operating parameters of the ant colony algorithm as the objects of optimization. According to experience, the range of pheromone factors *α* is generally [1,4], and the range of heuristic factors *β* is generally [3,5]. In this context, a genetic algorithm is employed to identify the optimal parameter values. The ant colony algorithm is integrated into the target optimization problem of the genetic algorithm, which is then executed. Additionally, the genetic algorithm repeatedly invokes the ant colony algorithm to refine the search for optimal parameters.

(3) Improvements to the Selection Strategy. After determining the transition probability of each node, the roulette algorithm is employed to select the next access node based on this probability. This approach ensures that ants do not prematurely lose diversity in their solutions during the path selection process. Additionally, by introducing a threshold mechanism, ants are guided to the next step of path selection only when the amount of information meets a specified threshold.


$$j= \{\begin{array}{c} \max\left\{\tau_{ij}^{a}(t)\cdot \eta_{ij}^{\beta }(t)\right\},j\in allowed_{k},\text{if}r\leq \rho^{0}\\ \text{Choose }j\text{ with probability }P_{ij}^{k},otherwise \end{array}$$ (17)

Where, $\rho^{0}\in �0�1�$, *r* is a uniformly distributed random number in (0,1).

This mechanism, after calculating the transition probability, introduces an element of randomness. Simultaneously, when the number of ants is substantial, it ensures that more ants will follow the current optimal route while allowing some ants to explore non-optimal but potentially promising paths. This randomness enhances the algorithm's global search capability and mitigates the tendency of the ant colony algorithm to converge on local optimal solutions.

(4) Improvement of Pheromone Renewal Strategy. Most traditional pheromone update methods utilize the ant week model. In this paper, an elite strategy is employed to update the pheromone. The pheromone is updated according to the following formula:


$$\tau_{ij}(t+1)=\rho \cdot \tau_{ij}(t)+\text{Δ}\tau_{ij}+\text{Δ}\tau_{ij}^{*}$$ (18)

Where, $\text{Δ}\tau_{ij}=\sum_{k=1}^{m}\text{Δ}\tau_{ij}^{k}$, $\text{Δ}\tau_{ij}^{k}$ see equation (12).


$$\text{Δ}\tau_{ij}^{*}= \{\begin{array}{cc} \sigma \cdot \frac{Q}{L^{*}} & \text{If edge }(i,j)\text{ is part of the optimal solution found}\\ 0 & otherwise \end{array}$$ (19)

Where, *σ* is the number of elite ants, $L^{*}$ is the optimal solution found, $\text{Δ}\tau_{ij}^{*}$ is the pheromone increment of elite ants to the path (i,j).

#### 2. Algorithm flow.

**Fig 3:**
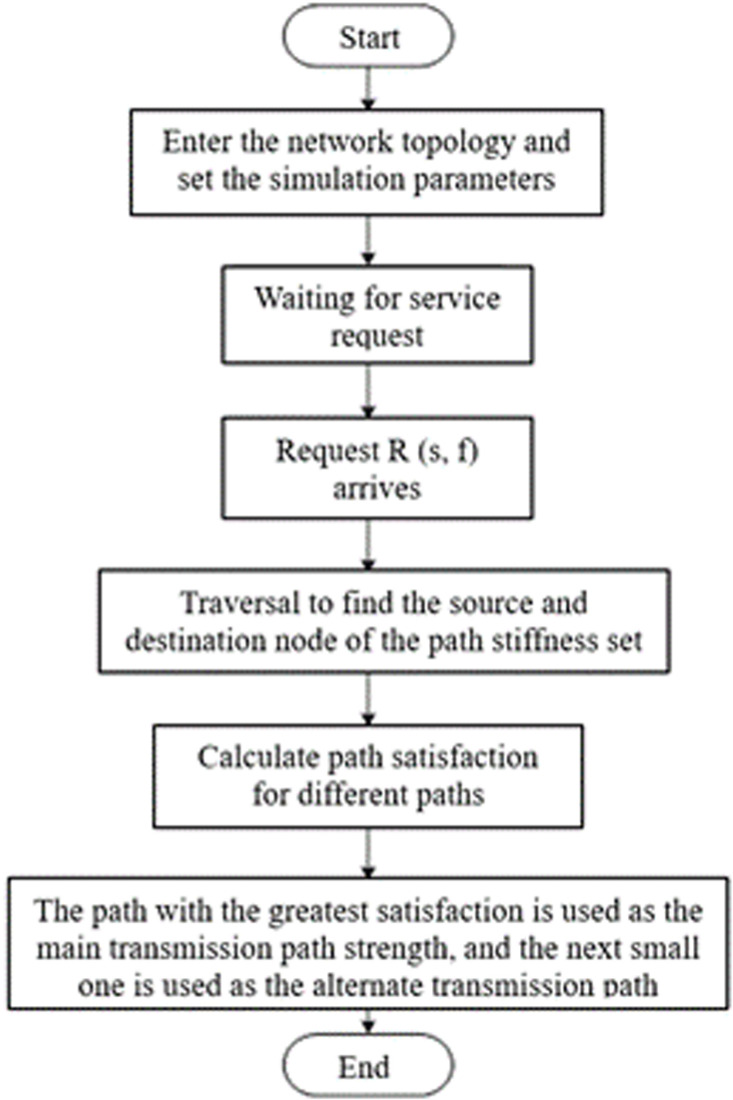
Routing policy based on path satisfaction.

The flowchart illustrating the specific algorithm implementation is presented in Fig 3, which primarily consists of three stages.

Step 1: Iterate to identify the set of paths from the source node to the destination node. All possible paths from the source node to the destination node are specified to obtain the set of candidate routes.

Step 2: Conduct a comprehensive evaluation and calculation of the satisfaction levels of various transmission paths, taking into account the network risk value and service forwarding delay.

Step 3: Based on the path satisfaction evaluation, select the path with the highest satisfaction as the primary real-time transmission path, and designate the second-best path as the alternate route.

### 3.3. Power optical communication network routing reconstruction algorithm based on service priority

In this section, we propose a service prioritization algorithm for reconstructing routing in power optical communication networks, specifically in scenarios where the active/standby communication routing link is faulty. Based on the fault state of the power optical communication network, we construct a hierarchical model for service differentiation, which allocates optimal transmission paths and resources for high-priority blocked services.

#### Algorithm description.

Literature [20] proposes a method for ranking service requests based on delay and reliability, prioritizing services with high resource demands and rapid response times. However, for video services, despite their significant resource requirements, they are considerably less critical than relay protection services. Therefore, based on the service importance evaluation results presented in Chapter 3, this section classifies services by priority according to their distinct characteristics. When designing the algorithm, two key issues must be addressed: the categorization of service levels and the allocation of resources. The prioritization of services is determined by the importance value assigned to each type of service, as evaluated in Chapter 3, to establish a reasonable recovery level for services. In the resource allocation process, high-priority services are granted preferential access to resources.

Therefore, services with similar performance requirements are grouped into the same priority category, with each priority level potentially encompassing multiple services. To streamline calculations, the numerous categories of service similarity importance are represented as positive integers within the range of [1,5]. In this context, smaller values correspond to higher priorities, as illustrated in Table 2. This section uses link faults as an example to design the algorithm. Assume that $B=\left\{b_{1},b_{2}\cdots b_{n}\right\}$ is the interrupted service set, *n* is the number of services, $b_{k}$ for Article *k* business. Each service $b_{k}$ is represented as a quadruple in the form of $\left(v_{k}^{s},v_{k}^{f},w_{k},l_{k}\right)$, Indicates the service start point, service end point, bandwidth requirement, and service priority.

**Table 2:** Business priorities.

Business name	Priority	Business name	Priority
500kV relay protection	1	Wide area pha-sor measurement	3
220kV relay protection	1	Lightning location monitoring	4
beat telephone	3	Substation video monitoring	4
dispatching automation	3	Administrative telephone	5
Stability system	2	Video conference	5

#### Algorithm flow.

**Fig 4:**
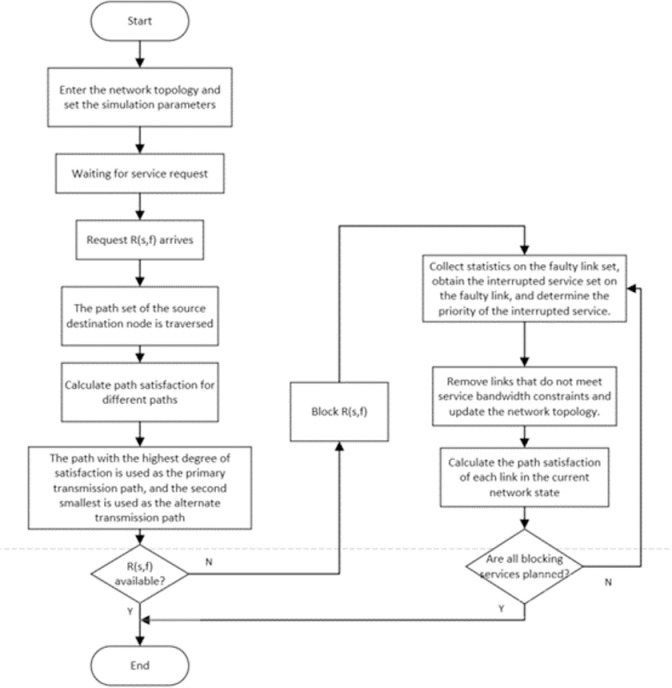
Routing reconstruction policy with service priority.

When the service channel is unavailable—specifically, when both active and standby routes are exhausted—set the trigger condition for the heavy route. Fig 4 illustrates the route reconstruction policy, which primarily consists of five stages. The algorithm flow is as follows:

Step 1: Collect statistics on the faulty link set and the interrupted service set, denoted as *F* and *D*,and determine the priority values of services in *D* according to the service priority division method in Table 2, and sort the priorities in ascending order.

Step 2: Traverse the faulty link set and reset the service $b_{k}(v_{s}^{k},v_{f}^{k})$ starting point with the highest priority on the faulty link $F(v_{s}^{m},v_{f}^{m})$ to $v_{s}^{m}$,and check whether the destination node $v_{f}^{k}$ of the service is the same as the other endpoint $v_{f}^{m}$ of *F*. If the destination node is the same and the node degree of $v_{f}^{m}$ is 0, it indicates that no valid line can share the services on the faulty line, and the service recovery fails. Update $D=D\backslash \left\{b_{k}\right\}$, Repeat Step 2. If the node degree of $v_{f}^{m}$ is not 0 or $v_{f}^{k}\neq v_{f}^{m}$, go to Step 3. If the service priorities are the same, a service is randomly selected for recovery.

Step 3: Remove links that do not meet service bandwidth constraints and update the network topology *G*. Calculate the satisfaction value of the alternative paths based on equation (8), and record the *K* paths with the highest satisfaction before service $b_{k}$ as $x_{1}(k),x_{2}(k),\cdots x_{k}(k)$, set l=1;

Step 4: Set the path $x_{l}(k)(1\leq l\leq K)$ that meets the constraints as the service $b_{k}$ recovery path, and update the available bandwidth of each link. If D=Φ, the algorithm ends. Otherwise, go to Step 5.

Step 5: Set l=l+1. If l�k, service recovery fails because no routes meet the restrictions. Otherwise, go to Step 4.

## 4. Simulation experiment

### 4.1. Experimental settings

**Fig 5:**
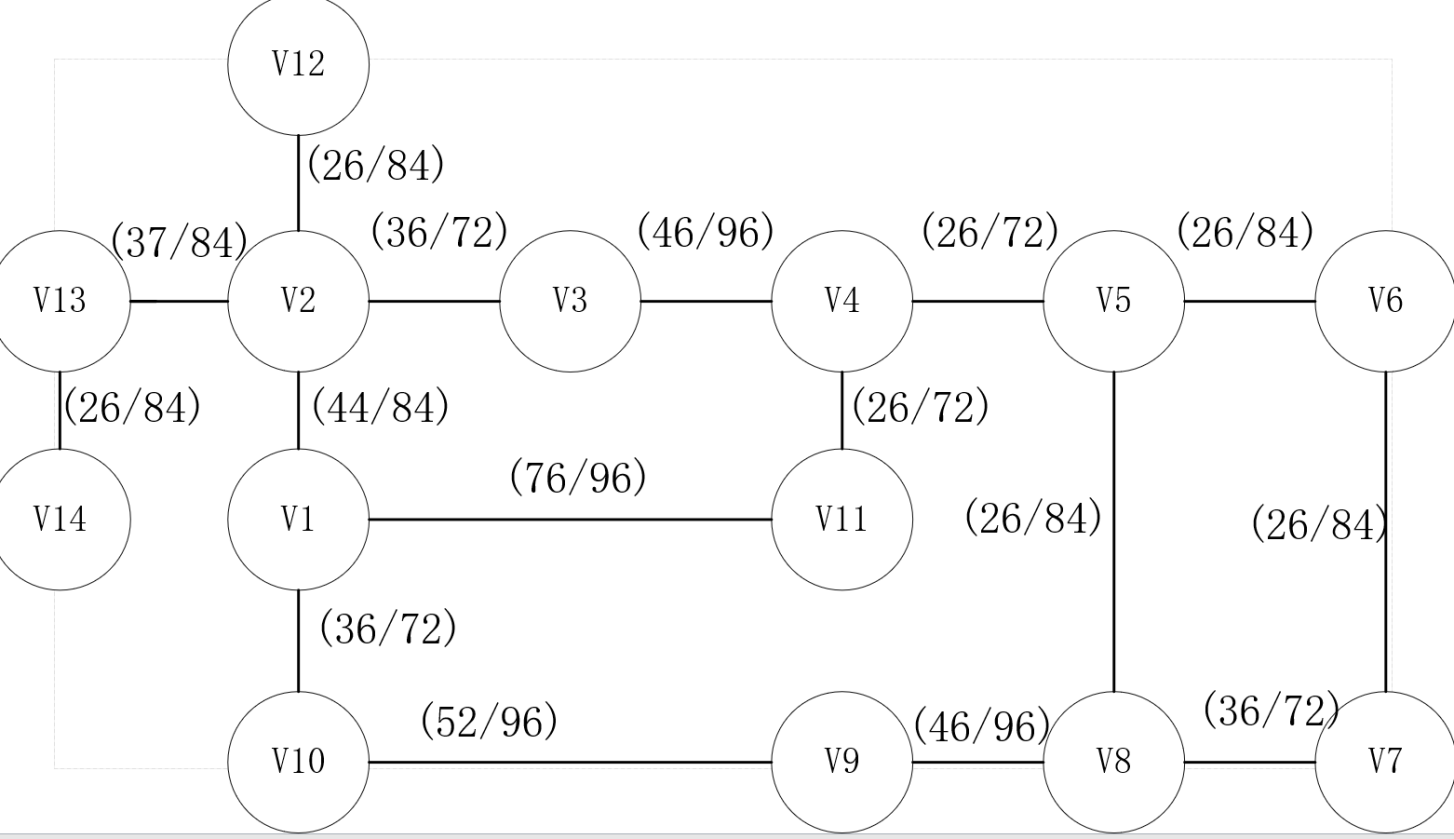
Topology of 14-node communication network.

In the simulation scenario, a 14-node communication network is analyzed (Fig 5). The network topology consists of 14 nodes and 16 links. Each node is identified by its corresponding number, while the array element adjacent to each node indicates the available capacity and total bandwidth resource of the link connecting the nodes, respectively. It is assumed that 10 types of services are distributed across the network, with the link bandwidth set to 1 Gbit/s. Services with varying priorities are generated according to a specified ratio. Given the generated routing request, a statistical analysis is conducted to evaluate the search performance of different policies.

### 4.2. Analysis of experimental results

#### Analysis of optimal scheduling results.

(1) Experimental parameter selection

**Fig 6:**
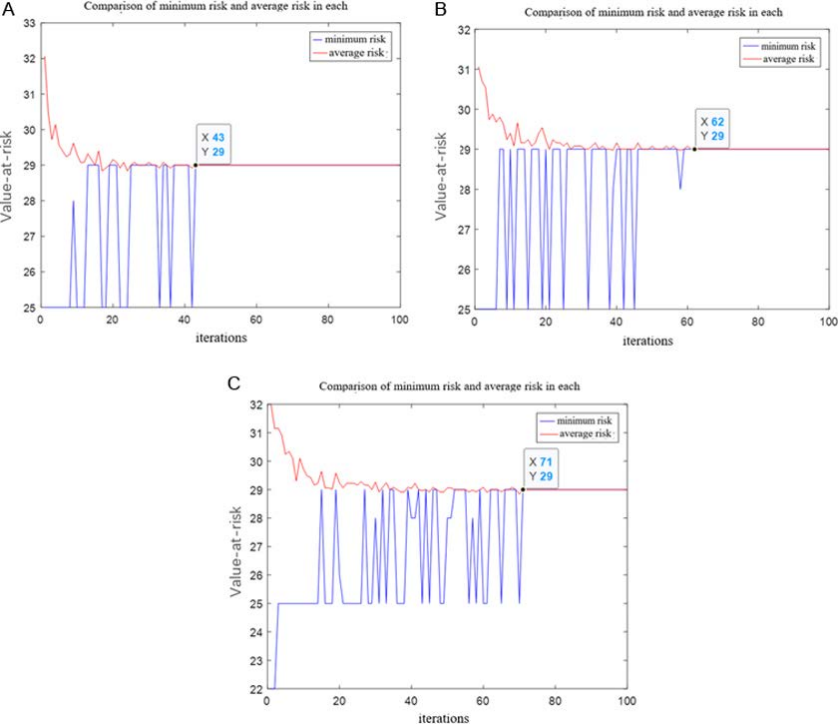
Changes of risk value under different parameters. (a) =1.1914, =3.4727 (b) =1, =5 (c) =4, =3.

Here, the output risk value is used as the evaluation index for selecting parameters (Fig 6).

We know that the competition between parameters *α* and *β* is that if *α* (pheromone importance) is too large, the heuristic value is almost ineffective, and the initial value of all the path pheromones is the same at the beginning, the algorithm is equivalent to ants walking randomly. If the value of *β* (the importance of the heuristic value) is too large, then every choice of the ant is almost determined by the path cost, and the algorithm is equivalent to a greedy strategy, and it is difficult to search for the global optimal solution. The optimized parameter values obtained in this paper are *α* = 1.1914 and *β* = 3.4727, and the number of iterations after optimization is more superior.

(2) Optimization result analysis

a. Optimization results

Based on the experimental results, this paper identifies the optimal and sub-optimal solutions from the routing optimization outcomes, designating them as the primary and alternate paths. Following route optimization, the active path is established as 1-11-4-5, while the standby path is designated as 1-2-3-4-5. The corresponding performance metrics are presented in Table 3. The term refers to the number of routers that services traverse.

**Table 3:** Performance of active and standby paths.

	Delay	Value-at-risk	Satisfaction	hop count
primary path	33.6056	29	0.4832	3
alternate route	33.6056	32	0.4529	4

According to the performance metrics of both the active and standby paths, it is evident that the active path outperforms the standby path, indicating that its Quality of Service (QoS) is superior.

When the active path becomes unavailable due to a fault, the system can swiftly transition to the standby path. The performance difference between the two paths is minimal. This standby switchover mitigates the impact of faults on the system and ensures service continuity during fault occurrences.

b. Traffic impact analysis

**Fig 7:**
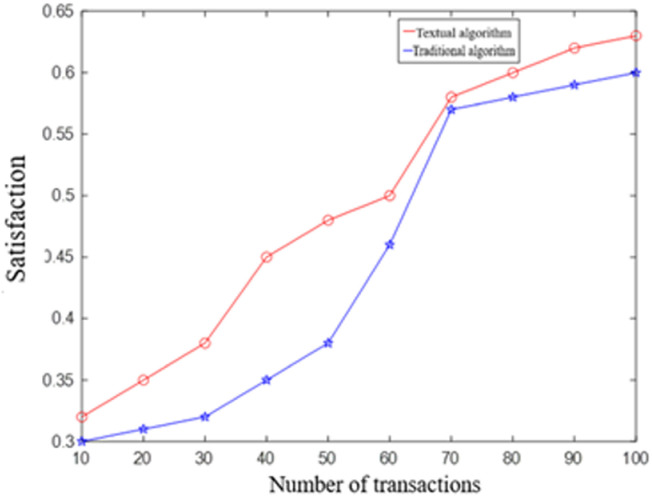
The relationship between the satisfaction and the number of transactions under the two methods.

Change the number of services distributed in the network and observe the planning results of the two methods (Fig 7).

It is evident from the results that as the number of services increases, the proposed algorithm consistently outperforms the traditional algorithm without enhancements. This demonstrates that the route selection algorithm, which is based on route satisfaction, remains unaffected by the number of services. Furthermore, the selected path consistently achieves an optimal balance between risk and delay.

c. Service priority verification experiment

**Fig 8:**
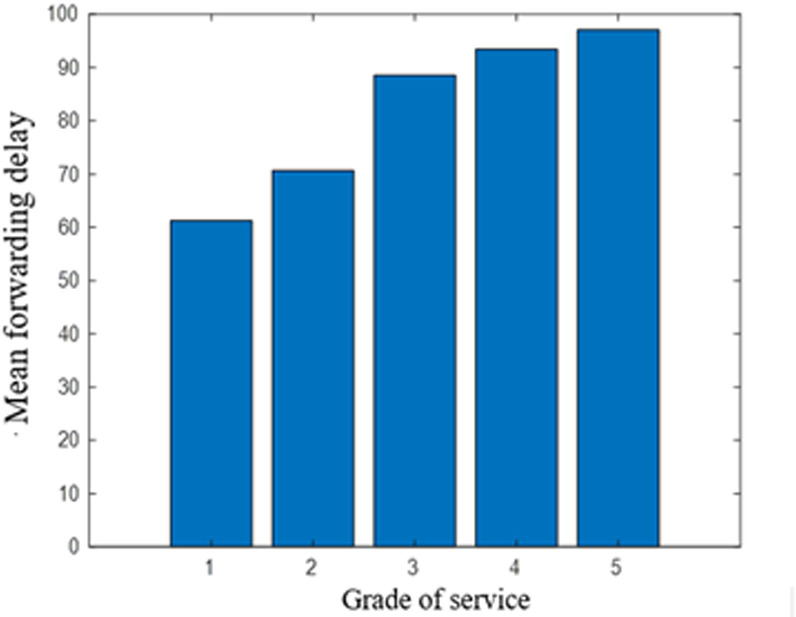
Comparison of forwarding delays for service recovery of different priorities.

When a common link fault occurs, and both the active and standby routes are unavailable, route recovery becomes necessary. The effect of service priority on service forwarding performance is illustrated in Fig 8.

The results indicate that as the priority of a service increases, the average packet forwarding delay decreases. This occurs because higher-priority services receive preferential treatment, resulting in a shorter queuing delay for these services. Thus, the effectiveness of the business priority classification method is demonstrated.

Therefore, in the event of an emergency, the routing re-optimization strategy based on service priority serves as a valuable reference for operational management. This strategy ensures the uninterrupted operation of critical services and optimizes the allocation of network resources to effectively address the impacts of emergencies, failures, or other unforeseen events.

### 4.3. Comparative experimental analysis

In order to verify the superiority of the proposed algorithm regarding Quality of Service (QoS) and the operational risk value of power optical communication networks, the proposed algorithm is compared with the traditional ant colony algorithm in terms of user satisfaction and service failure rate. The simulation results are presented below:

(1) Satisfaction comparison experiment

**Fig 9:**
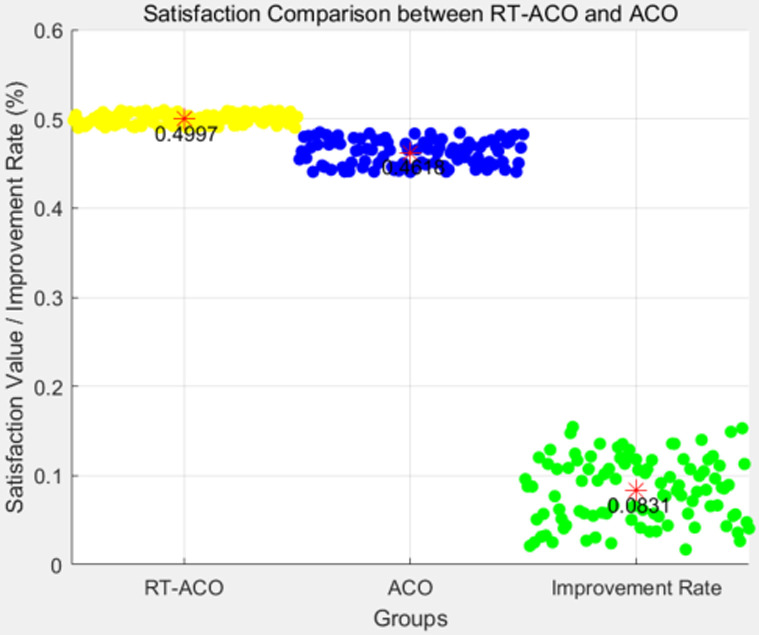
Comparison of Satisfaction between the proposed algorithm and the traditional ant colony algorithm.

As can be observed from the results shown in Fig 9, we conducted 100 experiments and recorded the satisfaction values of RT-ACO and ACO for each experiment in Fig 9, the satisfaction of the traditional algorithm converges to 0.4618 on average, while the satisfaction of the proposed algorithm converges to 0.4997 on average. The satisfaction of the proposed algorithm is 8.31% higher than that of the traditional algorithm. Thus, it is clear that the proposed strategy provides superior path performance compared to the conventional algorithms, indicating that the proposed approach outperforms the conventional ACO in terms of quality of service (QoS).

(2) Delay comparison experiment

**Fig 10:**
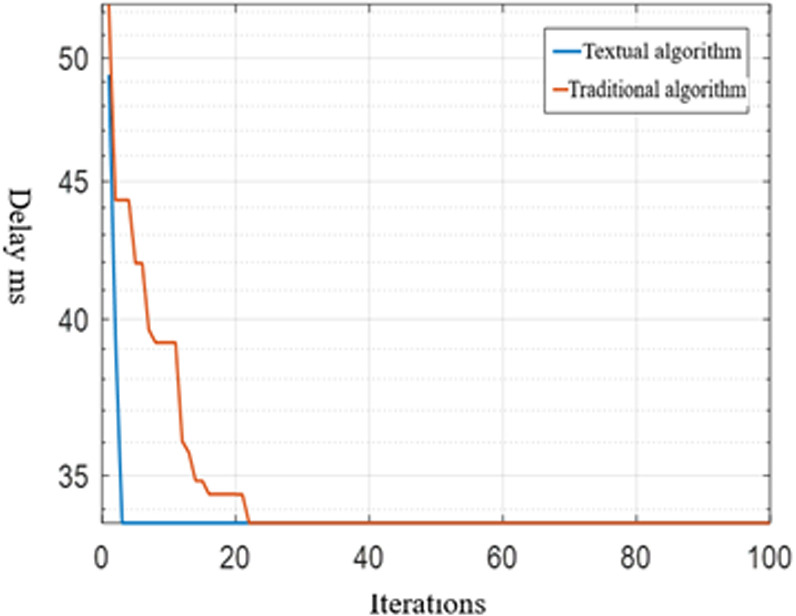
Changes of the delay of the two methods in iteration.

Fig 10 shows the change of the delay index. It can be seen that the algorithm in this paper converges quickly and the search process is stable in the iterative process. It can be seen that the proposed strategy can provide better path performance than traditional algorithms. It can be seen that the proposed strategy can provide better delay than the traditional algorithm, that is, the proposed method is better than the traditional ant colony algorithm in QoS.

(3) Comparative experiment of service failure rate

**Fig 11:**
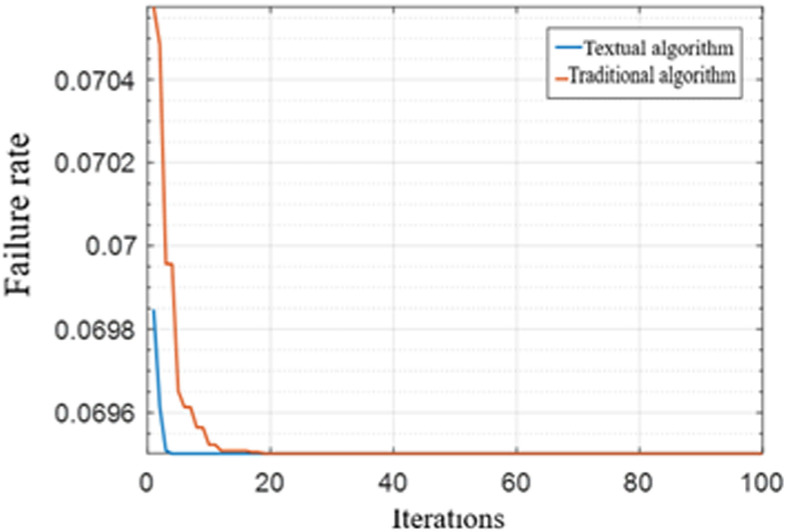
Comparison of service failure rate under the two methods.

To verify the efficiency of service failure management between the two methods, Fig 11 illustrates the changes in service failure efficiency between the proposed algorithm and the traditional algorithm during link failures. As shown in Fig 11, when both the primary routing path and its alternate routing path experience faults—triggering the rerouting condition—the improved algorithm converges more rapidly and effectively redistributes the services affected by the faulty line. In comparison to the traditional algorithm, the proposed method establishes a highly reliable rerouting path more swiftly. This indicates that the proposed strategy enhances network reliability beyond that of the traditional algorithm, demonstrating superior performance in terms of Quality of Service (QoS) compared to the traditional ant colony algorithm.

## 5. Conclusion

Focusing on the potential risks and scheduling challenges within power communication networks, this paper proposes an intelligent optimization method for business scheduling in power optical communication networks, emphasizing Quality of Service (QoS) guarantees. The proposed method establishes an objective function for routing optimization that comprehensively considers both the reliability of business transmission and associated time costs. It constructs a path satisfaction evaluation model based on risk and delay, and enhances the traditional ant colony algorithm in four key areas: the definition of heuristic information, the weighting of parameter values, the strategy for state selection, and the pheromone update strategy. By integrating risk information into the routing optimization process, this method offers a novel approach to routing optimization. Additionally, the algorithm can identify both the primary and backup routing paths for business transmission, prioritizing the transmission of high-priority tasks when both paths are unavailable. This optimization maximizes network service performance and minimizes the impact of partial information business interruptions on the operation and control of the power grid. Simulation results indicate a 7.4% improvement in path satisfaction compared to the traditional ant colony algorithm, demonstrating the feasibility and superiority of the proposed model and algorithm.

## Supporting information

S1 File DATA. (PDF)

S2 File DATA. (ZIP)

## Abbreviations

QoS: Quality of Service
ACO: Ant Colony Optimization
