# Supplementary material for: Intelligent tuning method for service scheduling in electric power communication networks based on operational risk and QoS guarantee
Source: PLoS One. 2025 Feb 24;20(2):e0317564. doi: 10.1371/journal.pone.0317564 (PMC11849824; doi:10.1371/journal.pone.0317564)
Supplement: S1 File — (PDF) [file pone.0317564.s001.pdf]

DATA

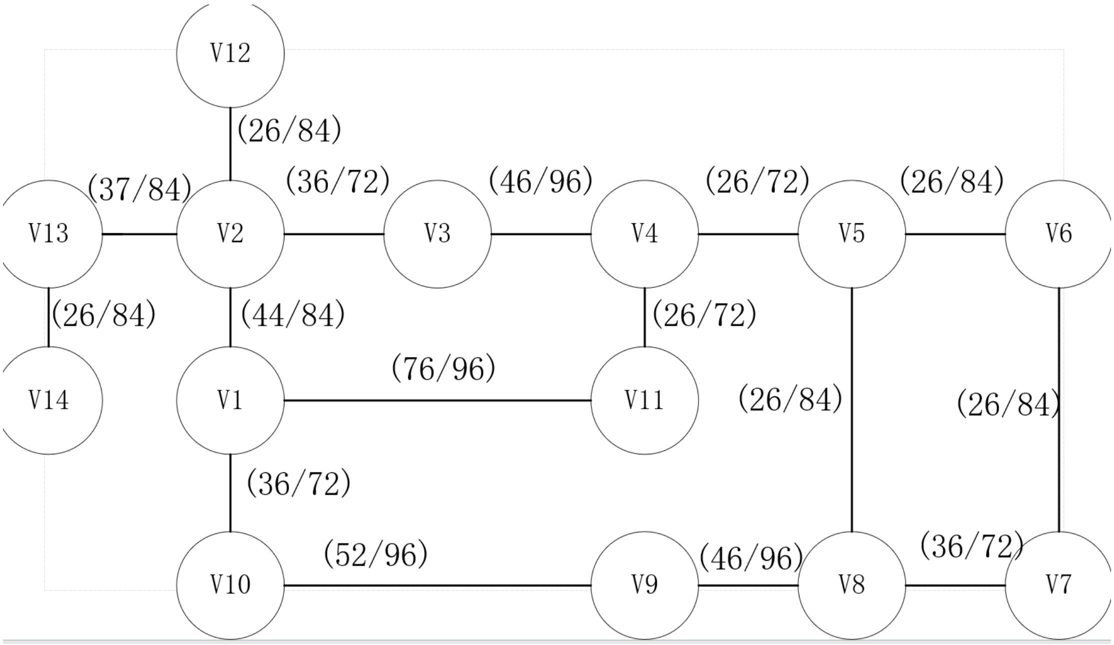

A 14-node communication network is analyzed. The network topology consists of 14 nodes and 16 links. Each node is identified by its corresponding number, while the array element adjacent to each node indicates the available capacity and total bandwidth resource of the link connecting the nodes, respectively. It is assumed that 10 types of services are distributed across the network, with the link bandwidth set to 1 Gbit/s. Services with varying priorities are generated according to a specified ratio. Given the generated routing request, a statistical analysis is conducted to evaluate the search performance of different policies.

Define the node data

```
nodes_data = {
    1, [2, 10, 11], [5, 3, 9], [12, 16, 14], [26, 36, 44], [84,72, 84];
    2, [1, 3, 12, 13], [2, 6, 4, 15], [12, 11, 5, 8], [37, 36, 26, 26], [84, 72, 84, 84];
    3, [2, 4], [5, 2], [7, 13], [36, 46], [72, 96];
```

```
    4, [3, 5, 11], [7, 4, 11], [13, 3, 9], [46, 26, 26], [96, 72, 84];
    5, [4, 6, 8], [3, 6, 12], [3, 8, 15], [26, 26, 36], [72, 72, 72];
    6, [5, 7], [5, 3], [8, 12], [26, 26], [72, 84];
    7, [6, 8], [8, 10], [12, 7], [26, 36], [84, 72];
    8, [5, 7, 9], [2, 5, 9], [15, 7, 14], [36, 46, 52], [72, 96, 96];
    9, [8, 10], [6, 3], [14, 8], [52, 46], [96, 96];
    10, [1, 9], [3, 12], [16, 8], [36, 52], [96, 96];
    11, [1, 4], [6, 5], [14, 9], [44, 26], [84, 84];
    12, [2], [4], [5], [26], [84];
    13, [2, 14], [5, 7], [8, 11], [26, 26], [84, 84];
    14, [13], [7], [11], [26], [84];
};
```

Define the node data (assume that each row corresponds to a node, column 1 is the node number, column 2 is the connected nodes, column 3 is the risk cost, column 4 is the delay, column 5 is the capacity, and column 6 is the total bandwidth resource).

Business Settings

```
% Each row: {Service Name, Priority, Bandwidth (Mbps), Delay Requirement (ms), Risk Tolerance}
services = {
    '500kV Relay Protection', 1, 100, 20, 5;
    '220kV Relay Protection', 1, 80, 30, 8;
    'Dispatch Phone', 3, 50, 50, 10;
    'Dispatch Automation', 2, 70, 40, 7;
    'Lightning Monitoring', 4, 30, 100, 15;
    'Video Conference', 5, 200, 150, 20;
};
```
