# Supplementary material for: Intelligent tuning method for service scheduling in electric power communication networks based on operational risk and QoS guarantee
Source: PLoS One. 2025 Feb 24;20(2):e0317564. doi: 10.1371/journal.pone.0317564 (PMC11849824; doi:10.1371/journal.pone.0317564)

**Code sharing declaration**

Due to privacy concerns, the code in this article cannot be open sourced. If you would like to receive data, please contact corresponding author Dou Zeng (17872506047@163.com) or contact information for non-authors (15725956055@163.com). The following contents are for the reference of editors and reviewers:

**Route optimization part：**

clc;

clear;

close all;

%% Initialize 14-node communication network topology data

nodes_data = {

1, [2, 10, 11], [5, 3, 9], [12, 16, 14], [26, 36, 44], [84, 96, 84];

2, [1, 3, 12, 13], [2, 6, 4, 15], [12, 11, 5, 8], [37, 36, 26, 26], [84, 72, 84, 84];

3, [2, 4], [5, 2], [7, 13], [36, 46], [72, 96];

4, [3, 5, 11], [7, 4, 11], [13, 3, 9], [46, 26, 26], [96, 72, 84];

5, [4, 6, 8], [3, 6, 12], [3, 8, 15], [26, 26, 36], [72, 72, 72];

6, [5, 7], [5, 3], [8, 12], [26, 26], [72, 84];

7, [6, 8], [8, 10], [12, 7], [26, 36], [84, 72];

8, [5, 7, 9], [2, 5, 9], [15, 7, 14], [36, 46, 52], [72, 96, 96];

9, [8, 10], [6, 3], [14, 8], [52, 46], [96, 96];

10, [1, 9], [3, 12], [16, 8], [36, 52], [96, 96];

11, [1, 4], [6, 5], [14, 9], [44, 26], [84, 84];

12, [2], [4], [5], [26], [84];

13, [2, 14], [5, 7], [8, 11], [26, 26], [84, 84];

14, [13], [7], [11], [26], [84];

};

%% Genetic Algorithm Parameters

pop_size = 30; % Population size

max_gen = 50; % Maximum generations

mutation_rate = 0.1; % Mutation rate

alpha_range = [1, 4]; % Range for alpha

beta_range = [3, 5]; % Range for beta

% Initialize the population as [alpha, beta]

pop = [rand(pop_size, 1) * (alpha_range(2) - alpha_range(1)) + alpha_range(1), ...

rand(pop_size, 1) * (beta_range(2) - beta_range(1)) + beta_range(1)];

%% Result Storage

best_fitness = zeros(max_gen, 1);

best_params = zeros(max_gen, 2);

best_paths = cell(max_gen, 1);

best_delays = zeros(max_gen, 1);

best_risks = zeros(max_gen, 1);

best_satisfactions = zeros(max_gen, 1);

second_best_paths = cell(max_gen, 1);

second_best_satisfactions = zeros(max_gen, 1);

second_best_delays = zeros(max_gen, 1);

second_best_risks = zeros(max_gen, 1);

%% Main Genetic Algorithm Loop

for gen = 1:max_gen

fitness = zeros(pop_size, 1);

paths = cell(pop_size, 1);

delays = zeros(pop_size, 1);

risks = zeros(pop_size, 1);

satisfactions = zeros(pop_size, 1);

% Evaluate the fitness of each individual

for i = 1:pop_size

alpha = pop(i, 1);

beta = pop(i, 2);

[fitness(i), paths{i}, delays(i), risks(i), satisfactions(i)] = ...

ACO(alpha, beta, nodes_data, 1, 5); % Call ACO algorithm

end

% Record the best path of the current generation

[best_fitness(gen), idx] = max(fitness);

best_params(gen, :) = pop(idx, :);

best_paths{gen} = paths{idx};

best_delays(gen) = delays(idx);

best_risks(gen) = risks(idx);

best_satisfactions(gen) = satisfactions(idx);

% Record the second-best path

temp_fitness = fitness;

temp_fitness(idx) = -Inf; % Exclude the best path

[second_best_fitness, second_idx] = max(temp_fitness);

second_best_paths{gen} = paths{second_idx};

second_best_delays(gen) = delays(second_idx);

second_best_risks(gen) = risks(second_idx);

second_best_satisfactions(gen) = satisfactions(second_idx);

% Selection (Roulette Wheel)

prob = fitness / sum(fitness);

cum_prob = cumsum(prob);

new_pop = zeros(size(pop));

for i = 1:pop_size

r = rand;

selected = find(r <= cum_prob, 1);

new_pop(i, :) = pop(selected, :);

end

% Crossover

for i = 1:2:pop_size-1

if rand < 0.8

cross_point = randi(2);

temp = new_pop(i, cross_point:end);

new_pop(i, cross_point:end) = new_pop(i+1, cross_point:end);

new_pop(i+1, cross_point:end) = temp;

end

end

% Mutation

for i = 1:pop_size

if rand < mutation_rate

mutation_idx = randi(2);

if mutation_idx == 1

new_pop(i, 1) = rand * (alpha_range(2) - alpha_range(1)) + alpha_range(1);

else

new_pop(i, 2) = rand * (beta_range(2) - beta_range(1)) + beta_range(1);

end

end

end

% Update the population

pop = new_pop;

end

%% Output Results

[best_fitness_val, best_gen] = max(best_fitness);

best_alpha = best_params(best_gen, 1);

best_beta = best_params(best_gen, 2);

best_path = best_paths{best_gen};

best_delay = best_delays(best_gen);

best_risk = best_risks(best_gen);

best_satisfaction = best_satisfactions(best_gen);

second_best_path = second_best_paths{best_gen};

second_best_satisfaction = second_best_satisfactions(best_gen);

second_best_delay = second_best_delays(best_gen);

second_best_risk = second_best_risks(best_gen);

fprintf('Optimal Parameters: alpha = %.2f, beta = %.2f\n', best_alpha, best_beta);

fprintf('Best Path Satisfaction: %.2f\n', best_satisfaction * 20);

fprintf('Best Path Delay: %.2f\n', best_delay);

fprintf('Best Path Risk: %.2f\n', best_risk);

fprintf('Best Path: %s\n', mat2str(best_path));

fprintf('Second Best Path Satisfaction: %.2f\n', second_best_satisfaction * 20);

fprintf('Second Best Path Delay: %.2f\n', second_best_delay);

fprintf('Second Best Path Risk: %.2f\n', second_best_risk);

fprintf('Second Best Path: %s\n', mat2str(second_best_path));

%% ACO Algorithm Implementation

function [fitness, best_path, delay, risk, satisfaction] = ACO(alpha, beta, nodes_data, start_node, end_node)

m = 50; % Number of ants

rho = 0.1; % Pheromone evaporation coefficient

Q = 1; % Pheromone constant

iter_max = 50;

% Initialize pheromone and heuristic factors

n = size(nodes_data, 1);

for i = 1:n

nodes_data{i, 5} = ones(1, length(nodes_data{i, 3})); % Pheromone

nodes_data{i, 6} = 1 ./ (nodes_data{i, 3} + nodes_data{i, 4}); % Heuristic factor

end

best_satisfaction = -Inf;

best_path = [];

best_delay = Inf;

best_risk = Inf;

paths = cell(m, 1); % Store paths for each ant

satisfactions = zeros(m, 1);

for iter = 1:iter_max

delays = zeros(m, 1);

risks = zeros(m, 1);

for ant = 1:m

current_node = start_node;

visited = [current_node];

delay = 0;

risk = 0;

while current_node ~= end_node

neighbors = nodes_data{current_node, 2};

neighbors(ismember(neighbors, visited)) = [];

if isempty(neighbors)

break;

end

% Calculate probabilities

probs = zeros(1, length(neighbors));

for k = 1:length(neighbors)

idx = find(nodes_data{current_node, 2} == neighbors(k));

probs(k) = (nodes_data{current_node, 5}(idx)^alpha) * ...

(nodes_data{current_node, 6}(idx)^beta);

end

probs = probs / sum(probs);

% Roulette wheel selection

cumulative_probs = cumsum([0, probs]);

r = rand;

next_node = neighbors(find(r >= cumulative_probs(1:end-1) & ...

r < cumulative_probs(2:end), 1));

% Update path, delay, and risk

visited(end + 1) = next_node;

idx = find(nodes_data{current_node, 2} == next_node);

delay = delay + nodes_data{current_node, 4}(idx); % Accumulate delay

risk = risk + nodes_data{current_node, 3}(idx); % Accumulate risk

current_node = next_node;

end

% Save path, delay, risk, and satisfaction for each ant

paths{ant} = visited;

delays(ant) = delay;

risks(ant) = risk;

satisfactions(ant) = calculateSatisfaction(visited, nodes_data, end_node);

end

% Record the best path

[max_satisfaction, idx] = max(satisfactions);

if max_satisfaction > best_satisfaction

best_satisfaction = max_satisfaction;

best_path = paths{idx};

best_delay = delays(idx);

best_risk = risks(idx);

end

% Pheromone Update (Elite Strategy)

for i = 1:m

path = paths{i};

for j = 1:length(path) - 1

current_node = path(j);

next_node = path(j + 1);

idx = find(nodes_data{current_node, 2} == next_node);

nodes_data{current_node, 5}(idx) = (1 - rho) * nodes_data{current_node, 5}(idx) + ...

Q / (risks(i) + delays(i));

end

end

end

% Return the best path and related information

fitness = best_satisfaction;

delay = best_delay;

risk = best_risk;

satisfaction = best_satisfaction;

end

%% Calculate Path Satisfaction

function satisfaction = calculateSatisfaction(path, nodes_data, end_node)

if path(end) ~= end_node

satisfaction = 0;

return;

end

Risk = 0;

Delay = 0;

for i = 1:length(path) - 1

current_node = path(i);

next_node = path(i + 1);

idx = find(nodes_data{current_node, 2} == next_node);

Risk = Risk + nodes_data{current_node, 3}(idx);

Delay = Delay + nodes_data{current_node, 4}(idx);

end

satisfaction = 1 / (Risk + Delay);

end

**Result：**

Output the optimal path and path information of the starting node：


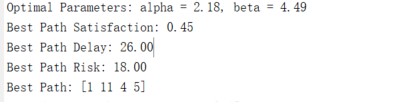


**Service scheduling part：**

clc;

clear;

close all;

%% Initialize the 14-node communication network topology

% Each row represents a node:

% {Node ID, Neighbors, Risk Cost, Delay, Remaining Capacity, Total Bandwidth}

nodes_data = {

1, [2, 10, 11], [5, 3, 9], [12, 16, 14], [26, 36, 44], [84, 96, 84];

2, [1, 3, 12, 13], [2, 6, 4, 15], [12, 11, 5, 8], [37, 36, 26, 26], [84, 72, 84, 84];

3, [2, 4], [5, 2], [7, 13], [36, 46], [72, 96];

4, [3, 5, 11], [7, 4, 11], [13, 3, 9], [46, 26, 26], [96, 72, 84];

5, [4, 6, 8], [3, 6, 12], [3, 8, 15], [26, 26, 36], [72, 72, 72];

6, [5, 7], [5, 3], [8, 12], [26, 26], [72, 84];

7, [6, 8], [8, 10], [12, 7], [26, 36], [84, 72];

8, [5, 7, 9], [2, 5, 9], [15, 7, 14], [36, 46, 52], [72, 96, 96];

9, [8, 10], [6, 3], [14, 8], [52, 46], [96, 96];

10, [1, 9], [3, 12], [16, 8], [36, 52], [96, 96];

11, [1, 4], [6, 5], [14, 9], [44, 26], [84, 84];

12, [2], [4], [5], [26], [84];

13, [2, 14], [5, 7], [8, 11], [26, 26], [84, 84];

14, [13], [7], [11], [26], [84];

};

%% Define service types and priorities

% Each row: {Service Name, Priority, Bandwidth (Mbps), Delay Requirement (ms), Risk Tolerance}

services = {

'500kV Relay Protection', 1, 100, 20, 5;

'220kV Relay Protection', 1, 80, 30, 8;

'Dispatch Phone', 3, 50, 50, 10;

'Dispatch Automation', 2, 70, 40, 7;

'Lightning Monitoring', 4, 30, 100, 15;

'Video Conference', 5, 200, 150, 20;

};

% Priority weight mapping: Higher priority gets more weight

priority_weight = [10, 8, 6, 4, 2];

%% Generate service flows

num_services = 10; % Number of service flows

service_flows = cell(num_services, 1); % Store [Source Node, Target Node, Bandwidth Demand]

for i = 1:num_services

service_type = randi(size(services, 1)); % Randomly select a service type

source_node = randi(14); % Randomly choose a source node

target_node = randi(14);

while target_node == source_node

target_node = randi(14); % Ensure the target node is different

end

service_flows{i} = [source_node, target_node, services{service_type, 3}];

end

%% Perform Ant Colony Optimization for each service

best_paths = cell(num_services, 1);

path_delays = zeros(num_services, 1);

path_risks = zeros(num_services, 1);

path_satisfactions = zeros(num_services, 1);

for i = 1:num_services

source_node = service_flows{i}(1);

target_node = service_flows{i}(2);

bandwidth_demand = service_flows{i}(3);

% Run ACO algorithm

[fitness, best_path, delay, risk, satisfaction] = ACO(2, 3, nodes_data, source_node, target_node);

% Store results

best_paths{i} = best_path;

path_delays(i) = delay;

path_risks(i) = risk;

path_satisfactions(i) = satisfaction;

% Update network link capacities

for j = 1:length(best_path) - 1

current_node = best_path(j);

next_node = best_path(j + 1);

idx = find(nodes_data{current_node, 2} == next_node);

nodes_data{current_node, 4}(idx) = nodes_data{current_node, 4}(idx) - bandwidth_demand;

end

end

%% Output results

disp('Service Scheduling Results:');

for i = 1:num_services

fprintf('Service %d: Source Node: %d, Target Node: %d\n', i, service_flows{i}(1), service_flows{i}(2));

fprintf(' Optimal Path: %s\n', mat2str(best_paths{i}));

fprintf(' Delay: %.2f ms, Risk: %.2f, Satisfaction: %.2f\n', path_delays(i), path_risks(i), path_satisfactions(i) * 20);

end

%% Ant Colony Optimization Function

function [fitness, best_path, delay, risk, satisfaction] = ACO(alpha, beta, nodes_data, start_node, end_node)

m = 50; % Number of ants

rho = 0.1; % Pheromone evaporation rate

Q = 1; % Pheromone constant

iter_max = 50;

% Initialize pheromones and heuristics

n = size(nodes_data, 1);

for i = 1:n

nodes_data{i, 5} = ones(1, length(nodes_data{i, 3})); % Pheromone

nodes_data{i, 6} = 1 ./ (nodes_data{i, 3} + nodes_data{i, 4}); % Heuristic info

end

best_satisfaction = -Inf;

best_path = [];

best_delay = Inf;

best_risk = Inf;

for iter = 1:iter_max

paths = cell(m, 1);

delays = zeros(m, 1);

risks = zeros(m, 1);

satisfactions = zeros(m, 1);

for ant = 1:m

current_node = start_node;

visited = [current_node];

delay = 0;

risk = 0;

while current_node ~= end_node

neighbors = nodes_data{current_node, 2};

neighbors(ismember(neighbors, visited)) = [];

if isempty(neighbors)

break;

end

% Calculate transition probabilities

probs = zeros(1, length(neighbors));

for k = 1:length(neighbors)

idx = find(nodes_data{current_node, 2} == neighbors(k));

probs(k) = (nodes_data{current_node, 5}(idx)^alpha) * ...

(nodes_data{current_node, 6}(idx)^beta);

end

probs = probs / sum(probs);

% Roulette wheel selection

cumulative_probs = cumsum([0, probs]);

r = rand;

next_node = neighbors(find(r >= cumulative_probs(1:end-1) & ...

r < cumulative_probs(2:end), 1));

% Update path, delay, and risk

visited(end + 1) = next_node;

idx = find(nodes_data{current_node, 2} == next_node);

delay = delay + nodes_data{current_node, 4}(idx); % Add delay

risk = risk + nodes_data{current_node, 3}(idx); % Add risk

current_node = next_node;

end

paths{ant} = visited;

delays(ant) = delay;

risks(ant) = risk;

satisfactions(ant) = calculateSatisfaction(visited, nodes_data, end_node);

end

% Update best path

[max_satisfaction, idx] = max(satisfactions);

if max_satisfaction > best_satisfaction

best_satisfaction = max_satisfaction;

best_path = paths{idx};

best_delay = delays(idx);

best_risk = risks(idx);

end

% Update pheromones

for ant = 1:m

path = paths{ant};

for j = 1:length(path) - 1

current_node = path(j);

next_node = path(j + 1);

idx = find(nodes_data{current_node, 2} == next_node);

nodes_data{current_node, 5}(idx) = (1 - rho) * nodes_data{current_node, 5}(idx) + ...

Q / (risks(ant) + delays(ant));

end

end

end

% Return best results

fitness = best_satisfaction;

delay = best_delay;

risk = best_risk;

satisfaction = best_satisfaction;

end

%% Satisfaction Calculation

function satisfaction = calculateSatisfaction(path, nodes_data, end_node)

if path(end) ~= end_node

satisfaction = 0;

return;

end

Risk = 0;

Delay = 0;

for i = 1:length(path) - 1

current_node = path(i);

next_node = path(i + 1);

idx = find(nodes_data{current_node, 2} == next_node);

Risk = Risk + nodes_data{current_node, 3}(idx);

Delay = Delay + nodes_data{current_node, 4}(idx);

end

satisfaction = 1 / (Risk + Delay);

end

**Result：**

The path optimization result of each service flow is output:


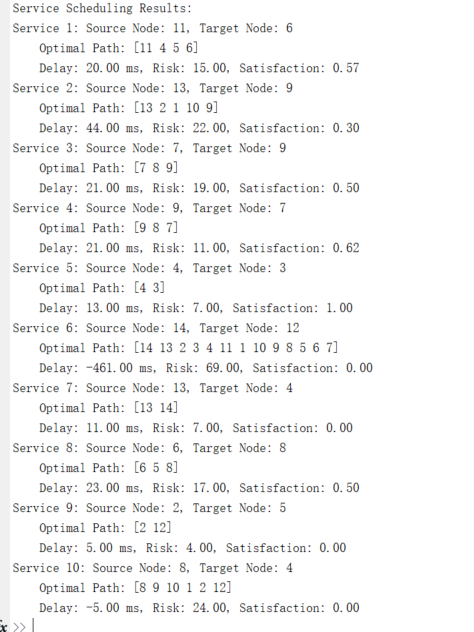

Supplement: S2 File — (ZIP) [file pone.0317564.s002.zip › supporting information/Code sharing declaration.docx]
