# Supplementary material for: Intelligent tuning method for service scheduling in electric power communication networks based on operational risk and QoS guarantee
Source: PLoS One. 2025 Feb 24;20(2):e0317564. doi: 10.1371/journal.pone.0317564 (PMC11849824; doi:10.1371/journal.pone.0317564)
Supplement: S2 File — (ZIP) [file pone.0317564.s002.zip › supporting information/Response to Reviewers.docx]

Dear Reviewers,

Thank you very much for your time involved in reviewing the manuscript 、your very encouraging comments on the merits and put forward very constructive suggestions.

Comments: *This paper proposes an intelligent tuning method of power optical communication network based on service scheduling and QoS guarantee. The relevant analysis and conclusion have certain value. But the following issues should be carefully addressed.*

We also appreciate your clear and detailed feedback and hope that the explanation below adequately addresses your concerns. In the rest of this letter, we will discuss each of your comments separately and our response accordingly.

To facilitate this discussion, we first retype your comments in italic font and then present our responses to the comments.

Comment 1-6:

*1.There is no section number throughout the paper.*

*2.The formulation of Eq. (8), Eq. (9), and Eq. (10) is ambiguous, e.g., “so the reliability model of power optical communication network is defined as formula (10):” followed by Eq. (8).*

*3.There are many formatting issues in the paper, e.g., what is the transition probability P_{ij}^{k} is proportional to? The expression does not match Eq. (12).*

*4.There is no punctuation at the end of the formula.*

*5.It should be Fig. not FIG. .*

*6.There are a lot of formatting errors in figures, e.g., Times New Roma font should be used.*

Response 1-6:

Thank you for the detailed review. We have carefully and thoroughly proofread the manuscript to correct all the grammar and typos.

Comment 7:

*7.What is Fig. 9 trying to say? Figure notes and axis labels don't match.*

Response 7:

Thank you for your question about the mismatch between the annotation of the drawing window and the axis label. We made a mistake in the title, which has been modified in the return draft.

Comment 8:

*8.Additional experiments should be added to illustrate ensuring QoS.*

Response 8:

Thanks for your great suggestion on improving the accessibility of our manuscript. As for the embodiment of QOS assurance in the experimental results, we have explained it in the article

Comments: *This paper presents an intelligent tuning method for power optical communication network based on service scheduling and QoS guarantee. In order to ensure the transmission of high-level services and optimize the service performance of the network to the greatest extent, this paper proposes a reconfiguration algorithm of power optical communication network based on service priority. The technical soundness and comprehensive contributions of the paper are clearly evident, as it explores the way to optimize the service performance of the network to the greatest extent, it provides a new direction for route search by considering the reliability of service transmission and the time cost.*

*1. Considering the characteristics of nodes in network physical topology, service bearing and resource utilization, a risk model is established.*

*2. Development of a route satisfaction evaluation function model with risk and delay as optimization objectives.*

*3. The RT-ACO (Risk-time aco) routing optimization algorithm is proposed, the heuristic function of ant colony model is improved, the algorithm parameters are optimized, and the selection strategy is improved.*

*4. Formulation of a route reconfiguration algorithm based on service priority to allocate ideal transmission paths and resources for high blocking services in power optical communication network.*

*While the paper presents substantial progress, a few areas need improvement:*

Response:

Thank you for taking the time to carefully review our article, sort out the general structure of this article, and put forward clear and valuable suggestions. I will reply to each of your suggestions in the following paragraphs, hoping to meet your requirements and make you satisfied.

Comment 1:

*1. Literature Review: The review needs to clearly articulate the distinctions and advancements offered by this work compared to existing studies. Specifically, the details of existing optimization algorithms should be discussed more extensively in this paper.*

Response 1:

Thank you for pointing out the shortcomings in the literature review of this paper. We have made adjustments in the revised draft and discussed the existing relevant studies more extensively and profoundly.

Comment 2:

*2. Mathematical Simplifications: To measure the impact of each risk factor, this paper defines a path satisfaction index. If the authors can simplify these expressions for some special cases, making the research results easier to understand and apply, it will be beneficial to the readers.*

Response 2:

Thank you for your suggestions on the mathematics of this paper to improve the readability of the paper. According to your suggestions, we have made adjustments in the revision draft to make the paper easier to understand.

Comment 3:

*3. Simulation Details: The paper could enhance its clarity by providing more detailed descriptions of the simulation parameters and scenarios. This would help verify the accuracy of the model and facilitate reproducibility.*

Response 3:

Thank you for your suggestions on the simulation of this paper. According to your suggestions, we have made adjustments in the revision draft, and made detailed modifications and detailed descriptions for the simulation part. For the convenience of readers.

Comment:

*Overall, the paper is technically sound and provides meaningful insights into the reliable operation of power optical fiber communication network systems. Addressing the above suggestions would strengthen the paper's impact and clarity.*

Response:

Thank you again for your valuable suggestions, we have made adjustments in the revision draft, and revised the grammar and accuracy of the whole article, hope to make you satisfied.

We would like to take this opportunity to thank you for all your time involved and this great opportunity for us to improve the manuscript. We hope you will find this revised version satisfactory.

Sincerely,

The Authors
